# Youth athletes sleep more, practice less, and may lose interest in playing sports due to social distancing mandates

**DOI:** 10.1016/j.pmedr.2022.101722

**Published:** 2022-02-02

**Authors:** Henry B. Ellis, Sophia M. Ulman, K. John Wagner, Connor M. Carpenter, Emily B. Gale, Kevin G. Shea, Philip L. Wilson

**Affiliations:** a Scottish Rite for Children, Center for Excellence in Sports Medicine, 2222 Welborn St, Dallas, TX 75219, USA; b University of Texas Southwestern Medical Center, Department of Orthopaedic Surgery, 5323 Harry Hines Blvd, Dallas, TX 75390, USA; c Scottish Rite for Children, Department of Psychology, 2222 Welborn St, Dallas, TX 75219, USA; d Stanford University School of Medicine, Department of Orthopaedic Surgery, 291 Compus Dr, Stanford, CA 94305, USA

**Keywords:** COVID-19, Youth Sports, Specialization, Psychological Health

## Abstract

In-person sport participation was suspended across the United States in the spring of 2020 to slow the spread of the novel coronavirus (COVID-19). The purpose of this study was to survey the impact of COVID-19 on young athletes during a period of social and organized sports restrictions. An anonymous cross-sectional survey study was conducted of youth athletes in the midst of social distancing mandates and consisted of six components: demographics, sport participation, changes in sport-related goals/aspirations, sleep habits, and measures of anxiety and depression. 711 individuals who accessed the survey link yielded 575 (81%) participants with responses available for analysis. All respondents (aged 13.0 years) played organized sports, 62% were single-sport athletes, and 74% considered high-level. Participants were training ∼3.3 h less per week, spending more time outside, and 86% of participants continued to train while social distancing. Sleep duration increased (∼1.2 h/night) and sleep quality improved in 29% of young athletes. Additionally, 22% and 28% reported PROMIS® anxiety and depression scores characterized as ‘mild’, ‘moderate’, or ‘severe’. Older single-sport participants reported higher depression scores, while higher anxiety scores were seen in female participants with fewer years played. 10% of young athletes and 20% of teenagers changed their sports-related goals. Training style modifications, decreased training, and increased sleep quantity and quality were positive effects of COVID-19 restrictions, while athletic aspirational changes were undesirable effects. Single-sport athletes may be at greater risk for psychological symptoms when their routine is altered.

## Highlights

• 62% were single sports athletes with >50% elementary and middle school aged.
• Specialization may contribute to psychological symptoms following routine alteration.
• Training hours per week decreased 3.3 h per week during the pandemic.
• Older participants, age 15–19 reported the least compliance with social distancing.

## 1 Introduction

In the US, an estimated sixty million children ages six to eighteen years participate in organized sport (DiFiori et al., 2014). However, following the first confirmed case of the novel coronavirus, 2019-nCoV (COVID-19), infection in the United States on January 20, 2020, social distancing guidelines shut down sports participation across the country (Holshue et al., 2020). Social distancing has been shown to reduce the spread of viruses such as influenza (Ahmed et al., 2018), and such measures may have played a pivotal role in past crises such as the SARS epidemic in 2003 (Wilder-Smith and Freedman, 2020). Based on a simulated mathematical model utilized in Singapore, Koo et al. suggested that a combination of quarantining infected individuals and social distancing might be employed to substantially reduce COVID-19 infections (Koo et al., 2020).

Evidence from past pandemics suggests that isolation and quarantine may be correlated with increased depression and anxiety (Hawryluck et al., 2004, Yu et al., 2017). Young athletes may be especially at risk due to the established link between sport participation and mental health (Biddle and Asare, 2011, Lubans et al., 2016, Rosenbaum et al., 2015). McMahon et al. surveyed 11,110 adolescents from ten European countries and found that frequent physical activity and participation in sport each contributed independently to decreased anxiety and depression (McMahon et al., 2017). In a 2013 systematic review of the psychological and social benefits of participation in sport for children and adolescents, Eime et al. proposed a model outlining the positive association of sport participation with psychological, psychosocial, and social health domains (Eime et al., 2013). Additional research agreed that team sport athletes may experience more mental health benefit than individual sport athletes, and incoming division I collegiate athletes demonstrate a lower prevalence of mental health conditions than non-athlete peers (Pluhar et al., 2019). These benefits are juxtaposed against the prevalence of early sport specialization and burnout amongst youth athletes. Year-round training in a single sport at early ages with added emphasis on high levels of competition and increased pressure to succeed is becoming increasingly common (Malina, 2010). When sport specialization occurs too early, athletes may experience negative impacts to both their physical and mental health (Brenner et al., 2016, Jayanthi et al., 2015).

Evaluating the ways in which young athletes have been uniquely affected by the drastic alteration of daily sport routines may provide a better understanding of the significant impact of sport participation on physical and mental well-being, the effects of mandated rest on current athlete culture, and provide data to guide treatment efforts for mental health concerns that may become more prevalent in the months following the implementation of social distancing mandates. Furthermore, knowledge of how youth athletes responded to the unexpected isolation during the pandemic may aide coaches, athletic trainers, and parents in addressing mental health in youth athletes during similar circumstances, such as a serious injury that could cause similar isolation. Additionally, insight into how youth athletes chose to spend their time while not under the pressure of highly competitive youth sports may provide perspective for how to supplement youth athletes’ time to potentially improve quality of life or, perhaps, to merely have more fun or get more sleep. The purpose of this study is to survey the physical impact, as well as mental health and sleep habits, of COVID-19 on young athletes and assess their psychological state during a period of social and organized sports restrictions. Our hypothesis is that youth athletes will likely continue training to a lesser degree for their athletic activity using modified techniques (i.e. virtual training). In addition, some athletes may lose interest in high level sports and may report symptoms associated with either depression or anxiety.

## 2 Methods

Approval from an Institutional Review Board was obtained prior to the initiation of this prospective survey study.

### 2.1 Survey design and distribution

An anonymous self-reported digital questionnaire was designed using REDCap electronic data capture tools hosted on an academic medical website and server to survey children and adolescents (Harris et al., 2009, Harris et al., 2019). The survey was sixty total questions consisting of six separate components: demographics, sport participation/training before and during COVID-19, changes in sport-related goals and aspirations, changes in sleep habits, Patient-Reported Outcomes Measurement Information System (PROMIS®) Emotional Distress – Anxiety – Pediatric Item Bank, and PROMIS® Emotional Distress – Depression – Pediatric Item Bank [PROMIS Health Organization (PHO) and PROMIS® Cooperative Group]. PROMIS® was developed to provide patient reported outcome measures (PROMs) with item banks across a variety of health domains (Cella et al., 2007). Eight non-disease specific PROMIS® short forms have been developed and tested in the pediatric population, among which are the anxiety and depression short forms (Walsh et al., 2008, Irwin et al., 2010). Due to the documented positive impact of sport participation on symptoms of anxiety and depression, and past evidence suggesting increased prevalence of these conditions during epidemics, these two short forms were selected for evaluating the psychological impact of COVID-19 on youth athletes (Hawryluck et al., 2004, Yu et al., 2017, McMahon et al., 2017). The publicly accessible survey link was distributed via a digital awareness campaign utilizing hospital mailing lists or through sports teams, community members, or organizations with which the Sports Medicine Department has a current or past relationship.

### 2.2 Study population

This study targeted athletes ages 6–19 across the United States. Before completing the survey, participants were asked to confirm their age and indicate consent (and assent for children ages 10–17) to participate in the study. Parents/Legal Guardians were asked to assist children under the age of 11 in completing the survey. Data was acquired from April 24, 2020 through May 12, 2020. This cutoff date was determined based on the initiation of staged lifting of social distancing measures (The White House CfDC, 2020). Participants that reported involvement in one or more sports were included.

### 2.3 Data analysis

Complete results from each individual component of the survey were included in analysis. Thus, the total sample size for the demographics component varied from the sample size analyzed for the PROMIS® components. The PROMIS® measures were scored on the T-score metric according to the guidelines set by the PROMIS® Health Organization (PHO) and PROMIS® Cooperative Group. Descriptive statistics were calculated for all measures across the full sample, as well as by age and gender. Specifically, trends by age were highlighted by dividing the full sample into three age groups (Group 1: 6–9 years, Group 2: 10–14 years, and Group 3: 15–19 years). For paired comparisons, including hours of training and sleep before verses during COVID-19, Wilcoxon signed-rank tests were performed. Additionally, Kruskal-Wallis tests were performed to identify differences across both depression and anxiety groups (i.e., none to slight, mild, and moderate/severe) for age, gender, specialization status, altered aspirations, and changes in sleep duration and quality, followed by Mann-Whitney U tests for those with statistical significance. For all statistical comparisons with *p* < 0.05, the finding was considered statistically significant.

## 3 Results

### 3.1 Demographics

A total of 711 surveys were returned, with 575 (80.9%) meeting inclusion criteria. On average, survey participants were 13.0 years of age. The majority of those sampled had played organized sports for multiple years, and reported participation in sports 42.1 weeks/year. Additionally, 73.6% were considered high-level athletes (competing at a higher level than recreational or school sports) (Table 1). 62.4% of all participants were single-sport athletes with >50% of elementary and middle school aged kids reporting that they play a single sport, which indicated a high incidence of early sport specialization in this cohort.

**Table 1:** Demographics and sports participation by age and gender.

	All	Age			Gender	
		Group 1	Group 2	Group 3	Female	Male
N	575 (M, F)	58 (36, 22)	324 (127, 197)	193 (86, 107)	326	249
Range (years)	6–19	6–9	10–14	15–19	6–18	6–19
Mean Age ± SD (years)	13.0 ± 2.8	7.8 ± 1.0	12.1 ± 1.4	16.1 ± 1.1	13.1 ± 2.6	12.9 ± 3.1
Total Yrs Played (M ± SD)	7.5 ± 3.4	3.8 ± 1.2	7.2 ± 2.3	9.0 ± 3.9	7.1 ± 3.1	7.7 ± 3.4
Active Wks/Yr (M ± SD)	42.1 ± 11.3	38.2 ± 13.3	43.6 ± 10.3	41.1 ± 11.5	44.7 ± 9.5	39.4 ± 12.2
Single-Sport Athletes (%)	62.4	55.2	58.6	71.0	66.0	57.8
High-Level (%) €	73.6	72.4	80.3	62.7	73.6	73.5

€ High level athlete was established when a respondent answered that their highest level of competition was more than at the recreational or school level.

### 3.2 Effect on training

Prior to the pandemic, participants were training 9.7 h per week on average. During the pandemic, training significantly reduced to 6.4 h per week (*p* < 0.01) (Table 2). 86.2% of participants continued to train while social distancing, and the majority of participants maintained communication with their sports team (76.0%). Of those that continued training, 70.6% reported participating in self-selected workouts, 66.3% in coach-provided workouts, and 40.1% in virtual group workouts.

**Table 2:** Change in training hours/week and sleep hours/night.

	Pre- COVID-19	During COVID-19	*p*-value
**Training (hours/week)**			
All	9.7 ± 7.4 (0–42)	6.4 ± 5.1 (1–38)	**<0.001**
Age Group 1	6.4 ± 5.4 (1–23)	5.5 ± 4.6 (1–20)	
Age Group 2	8.7 ± 7.3 (0–42)	5.8 ± 4.8 (1–30)	
Age Group 3	12.4 ± 7.2 (0–38)	7.8 ± 5.5 (1–38)	

**Sleep (hours/night)**			
All	7.9 ± 1.3 (4–11)	9.1 ± 1.4 (2–13)	**<0.001**
Age Group 1	9.2 ± 1.0 (7–11)	9.7 ± 1.0 (7–12)	
Age Group 2	8.2 ± 1.2 (4–11)	9.3 ± 1.3 (4–13)	
Age Group 3	7.1 ± 1.2 (4–10)	8.8 ± 1.6 (2–13)	
*Significant differences are bolded.*			

### 3.3 Effect on wellness and socialization

During the pandemic, 47.1% of participants spent more time outside, with Groups 1 and 2 reporting ‘more’ or ‘a lot more’ time outside (53.7% and 52.8%, respectively), while 64.6% of Group 3 spent less or the same amount of time outside. The majority of respondents (64.9%) reported ‘always’ adhering to social distancing. However, the older adolescents, Group 3, were most likely to break social distancing as 45.2% reported not ‘always’ adhering to guidelines, 72.5% of which reported still spending time with their friends.

Overall, the reported average hours of sleep per night significantly increased from 7.9 h pre-COVID-19 (range 4–11) to 9.1 h (range 2–13) during the pandemic (*p* < 0.01) (Table 2). Increased sleep duration and quality was most reported in the older age group and trended inversely by age.

### 3.4 Effect on mental health and sports aspirations

Approximately one quarter of pediatric and adolescent respondents to the survey reported elevated depression scores during the pandemic. 28.3% of depression scores were identified as ‘mild’, ‘moderate’, or ‘severe’; and 22.2% of participants reported some degree of anxiety. Furthermore, 13.3% reported a change in goals or aspirations due to the pandemic; with the majority of those feeling they had lost opportunities to compete at a higher level (52.8%) or lost interest in training hard (41.7%). Additionally, Group 3 was more likely to report changes in sport-related goals and aspirations (23.6% verses < 9% in Groups 1 and 2).

Respondents in the ‘moderate/severe’ depression group reported their sport-related goals changed more frequently than the ‘none to slight’ depression group (*p* = 0.02) citing loss of interest in training hard (73.3%) as the most common reason (Table 3). Athletes with ‘moderate/severe’ depression scores were more likely to report worsened sleep quality during the pandemic compared to both‘none to slight’ and ‘mild’ groups (*p* < 0.01). Also, those with ‘none to slight’ depression scores, when compared to respondents with ‘mild’ or ‘moderate/severe’ scores, were more likely to report participation in multiple sports (*p* = 0.02). Similarly, any change in sleep quality or duration, whether worsened or improved during the pandemic, was associated with elevated anxiety scores (*p* < 0.01 and *p* = 0.02, respectively). Lastly, females made up a disproportionate percentage of the ‘moderate/severe’ (73.6%) and ‘mild’ (60.9%) anxiety groups.

**Table 3:** Differences between PROMIS® depression and anxiety severity score.

**Depression**	None to slight (n = 349)	Mild (n = 66)	Moderate/Severe (n = 70)	*p*-value
Age (yrs)	12.79 ± 2.66	13.48 ± 3.04	14 ± 2.87^N^	**0.001**
Gender (Male)	45.0	34.8	34.3	0.113
Single-Sport Athlete	59.6	65.2	77.1^N^	**0.020**
Changed Aspirations	10.6	18.2	21.4^N^	**0.022**
Change in sleep (hrs)	1.5 ± 1.77	1.95 ± 1.81	1.15 ± 2.27	0.164
Sleep Quality				**<0.001**
Improved	30.1	25.8	32.9	
Stayed the same	55.3	53.0	25.7	
Worsened	14.3	21.2	41.4	

**Anxiety**	None to slight (n = 364)	Mild (n = 64)	Moderate/Severe (n = 53)	*p*-value
Age (yrs)	13 ± 2.8	13.17 ± 2.94	13.42 ± 2.61	0.640
Gender (Male)	44.8	39.1	26.4^N^	**0.036**
Single-Sport Athlete	60.2	70.3	71.7	0.110
Changed Aspirations	12.6	17.2	13.2	0.613
Change in sleep (hrs)	1.62 ± 1.79	0.71 ± 2.06 ^N^	1.54 ± 2.11	**0.024**
Sleep Quality				**<0.001**
Improved	30.5	32.8	26.4	
Stayed the same	55.2	37.5	32.1	
Worsened	14.0	29.7	41.5	
*Continuous variables represented as Mean ± SDCategorical variables represented as Percentage of Comparison groupSignificant differences (α = 0.017) noted with a superscript ^N^ or ^M^ to indicate differences from ‘None to slight’ and ‘Mild’, respectively.*				

## 4 Discussion

A survey was conducted to specifically investigate the response of youth athletes during a modern-day epidemic. A true pandemic, such as COVID-19, has resulted in decreased organized training, more sleep, and more time outdoors by surveyed youth athletes. While this may be interpreted by some, and perhaps the athletes themselves, as potentially positive changes; the restrictions and resultant social upheaval may have resulted in sleep degradation or depressive symptoms in others.

The imposed social distancing restrictions resulted in various strategies to continue training. Self-selected workout and coach-provided self-paced workout schedules were followed by more than half of the respondents, and 40% participated in virtual group sessions. Although there is limited knowledge of the effect of virtual training in youth sports, live virtual training has been established as a well-accepted education tool in medical education (Umoren et al., 2017, Hudlicka, 2013). In addition to reduced volume of training, these new techniques of varied training programs, including live virtual training, may be considerations to reduce loads and time commitments of in-person team training in the future.

Another potential effect of the mandated social distancing that resulted in a freeze on youth sports activity may be the reduction in burnout risk for some athletes. With an estimated 70% of kids quitting organized sports in middle school, a healthy rest from the intensity of practice, over-scheduled travel weekends, and the (over)emphasis placed on winning by parents and coaches may serve as a breath of fresh air for some young athletes during this time (Maruyama et al., 2018, Aunola et al., 2018). Lastly, while organized training time was decreased, respondents in the younger two groups reported spending more time outside than usual. This may demonstrate more of an opportunity for free play or unstructured outdoor play which is felt to be beneficial in these early years.

Sleep quality and quantity have recently been established as important off the field considerations in youth athletes (Potter et al., 2020, Garmy and Ward, 2018). Milewski et al. (Milewski et al., 2014) in a review of 160 student athletes (mean age 15 years old), reported that athletes with less than eight hours of sleep per night were 1.7 times (CI 1.0–3.0, *p* = 0.04) more likely to sustain a musculoskeletal injury. This finding was later supported by a systematic review of five studies with similar findings (Gao et al., 2019). In a study of adolescents with concussions, poor sleep quality was associated with greater symptom severity and prolonged recovery (Chung et al., 2019). Implications of poor sleep habits beyond musculoskeletal injury may include increasing pain, tiredness, and symptoms associated with mood disorders (Garmy and Ward, 2018, Andreucci et al., 2020). Improved sleep has also been reported to be a protective mechanism against depression in the adolescent (Gangwisch et al., 2010).

In the current survey, the average hours of sleep per night increased from a reported 7.9 h pre-COVID-19 to 9.1 h during COVID-19 social distancing restrictions. Notably, the sleep quantity was reported to be increased in approximately half of those in the younger two age groups, while the older teens reported increased sleep duration from baseline >60% of the time. While further investigation is required to determine clear relationships, it seems likely that decreased demands of the usual school and concurrent organized sports schedules may result in more and improved sleep for many children and adolescents.

The benefits of sport participation on mental health in children and adolescents has been well-documented (McMahon et al., 2017, Eime et al., 2013). One in four respondents to this survey reported depression scores identified as ‘mild’, ‘moderate’, or ‘severe’; and one in five pediatric and adolescent athletes reported some degree of anxiety during the COVID-19 pandemic. Whether these levels are a direct result of the social and activity level changes imposed on sports participation during the pandemic, and how they may have differed from pre-pandemic levels, is beyond the scope of this study. However, contrary to adult elite athletes (Rice et al., 2016), self-reported PROMIS® depression and anxiety scores across all ages in pediatric and adolescent athletes have been previously reported to be 8% (Pluhar et al., 2019, Makhni et al., 2019). Although direct causation with either depression or anxiety cannot be established, PROMIS® depression and anxiety scores in this survey may raise awareness of the possibility of developing mental health concerns in these age groups during the pandemic.

Recommendations against sports specialization (playing one sport more than eight months of the year) and excessive sports volume (participation in organized sport totaling more hours per week than age of the athlete) have been established to reduce single event and overuse injuries (Jayanthi et al., 2015, Fabricant et al., 2016, Bell et al., 2018). This survey indicates that 62.4% of respondents were single-sport athletes, and a 34.0% reduction in training hours was reported. Although no consensus has been reached and multi-sport participation beyond youth sports has been advocated (Rugg et al., 2018), most have suggested avoiding sports specialization until at least 14 years of age (Bell et al., 2018, Myer et al., 2015). This survey further highlights the concerning national trend of sports specialization at younger ages, with >50% of those less than 15 years old participating in a single sport.

13% of these youth athletes reported a change in goals or aspirations due to the pandemic. This was reported in nearly a quarter of athletes in the older adolescent group. Of those reporting these aspirational changes, over 40% also reported depression scores above the ‘none to slight’ level. Finally, the direct effect of altered opportunity to compete and advance within their chosen sport seems to have affected many of those that reported alterations in goals and aspirations; with over 40% reporting that they felt they had lost opportunities to compete at a higher level or lost interest in training.

Some additional findings suggesting particular youth athletes at risk for PROMIS® depression and anxiety scores were also notable. Specifically, the possibility that the older specialized athlete may be at risk for depressive and anxiety symptoms in this unique setting of the removal of sports participation merits further study. The effects of the pandemic across a broad population may provide insight into some challenges facing these individual youth athletes when injury, aging out, or other attritional causes lead to a loss of sports structure and identity.

This study and survey are not without limitations. The associations noted in the youth athletes sampled may be subject to regional and sampling bias as a result of connection to a particular pediatric sports medicine center. The limited data on baseline mood disorders and PROMIS® depression and anxiety scores impacts the interpretation of the effect of COVID-19 on these findings. Although the PROMIS® questionnaires have previously been validated, additional survey questions used in this survey were uniquely developed and thus psychometric properties were not tested or evaluated. This was in part due to the timely nature of the distribution of the survey during social restrictions mandates but does highlight a significant limitation in a survey of this nature. Additionally, in order to maximize compliance with completion of the survey, the investigators focused on a limited data set. Items such as underlying medical conditions, medication, individual educational modifications, and economic stress were not evaluated, but may have implications on the responses of the participants. Lastly, the investigators acknowledge that many comparisons were performed. While the *p*-values were not adjusted for multiple tests, raw values were provided such that readers may adopt the Bonferroni adjustment, if needed.

## 5 Conclusion

In conclusion, the imposed restrictions surrounding the COVID-19 pandemic may have resulted in both beneficial and negative effects on high-level youth athletes. Young athletes, on average, spent more time outside and slept more hours per night. However, older, single-sport athletes tended to have elevated PROMIS® depression scores. Over one in ten young athletes and one in five teenagers changed their sports related goals and aspirations due to the COVID-19 restrictions.

## CRediT authorship contribution statement

Henry Ellis, Sophia Ulman, and John Wagner were involved in conceptualization, methodology, and writing. Sophia Ulman also performed formal analysis of the data. All authors contributed to some aspect of writing the manuscript, whether writing the orginial draft or primarily reviewing and editing.

## Declaration of Competing Interest

The authors declare that they have no known competing financial interests or personal relationships that could have appeared to influence the work reported in this paper.
